# Acidification Activates *Toxoplasma gondii* Motility and Egress by Enhancing Protein Secretion and Cytolytic Activity

**DOI:** 10.1371/journal.ppat.1004488

**Published:** 2014-11-06

**Authors:** Marijo S. Roiko, Nadezhda Svezhova, Vern B. Carruthers

**Affiliations:** Department of Microbiology and Immunology, University of Michigan Medical School, Ann Arbor, Michigan, United States of America; University of Wisconsin Medical School, United States of America

## Abstract

Pathogenic microbes rely on environmental cues to initiate key events during infection such as differentiation, motility, egress and invasion of cells or tissues. Earlier investigations showed that an acidic environment activates motility of the protozoan parasite *T. gondii*. Conversely, potassium ions, which are abundant in the intracellular milieu that bathes immotile replicating parasites, suppress motility. Since motility is required for efficient parasite cell invasion and egress we sought to better understand its regulation by environmental cues. We found that low pH stimulates motility by triggering Ca^2+^-dependent secretion of apical micronemes, and that this cue is sufficient to overcome suppression by potassium ions and drive parasite motility, cell invasion and egress. We also discovered that acidification promotes membrane binding and cytolytic activity of perforin-like protein 1 (PLP1), a pore-forming protein required for efficient egress. Agents that neutralize pH reduce the efficiency of PLP1-dependent perforation of host membranes and compromise egress. Finally, although low pH stimulation of microneme secretion promotes cell invasion, it also causes PLP1-dependent damage to host cells, suggesting a mechanism by which neutral extracellular pH subdues PLP1 activity to allow cell invasion without overt damage to the target cell. These findings implicate acidification as a signal to activate microneme secretion and confine cytolytic activity to egress without compromising the viability of the next cell infected.

## Author Summary

*Toxoplasma* and related parasites including those that cause malaria are obligate intracellular pathogens that replicate within a specialized compartment termed the parasitophorous vacuole. To infect new host cells these parasites must first escape from the parasitophorous vacuole and other limiting membranes of the currently infected cell. Escape, or egress as it is often called, depends on the timely release of adhesive proteins and lysis factors from secretory organelles called micronemes. Although this secretory event is crucial for egress, the natural environmental cues that trigger microneme secretion remain poorly defined. Here we discover that acidification of the parasitophorous vacuole is sufficient to trigger microneme secretion and promote the activity of a lysis factor called PLP1. We also show that pH-neutralizing drugs inhibit egress and provide evidence of parasitophorous vacuole acidification approximately coinciding with parasite egress from infected host cells. The findings support a working model in which acidification activates microneme dependent motility and lytic activity to execute egress and destruction of infected cells. The results also provide insight into how PLP1 lytic activity is stimulated during egress in an acidic environment and subsequently suppressed by the neutral extracellular environment, thus permitting cell invasion with minimal damage to the next target cell.

## Introduction

Infectious microorganisms experience diverse microenvironments during infection of a host. Such pathogenic microbes utilize specific cues to assess the environment and trigger an appropriate response that aids in their survival. For example, malaria parasites (*Plasmodium* spp) sense xanthurenic acid and a drop in temperature upon infecting a mosquito to trigger male gamete exflagellation for sexual reproduction [1]. The related apicomplexan parasite *Toxoplasma gondii* is thought to utilize the rapid build up of self-made abscisic acid as an intrinsic cue to exit from infected cells [2], an event termed egress. However, other studies have shown that *T. gondii* can respond to diverse environmental changes including the loss of host cell viability [3], [4] or an increase in reduction potential [5] to trigger egress. Most apicomplexan parasites including, malaria parasites and *T. gondii*, replicate within a membrane bound compartment termed the parasitophorous vacuole (PV). The PV microenvironment is presumed to be similar to that of the host cell cytoplasm based on the detection of a hypothetical pore that permits fluorescent dyes of <∼1,300 Da freely pass across the PV membrane [6]–[8]. However, the identity of this pore has not been reported for malaria parasites or *T. gondii*. Also, the malaria PV has much higher Ca^2+^ concentrations (∼40 µM) than the cytosol of infected erythrocytes (100 nM) [9], indicating a restricted flow of Ca^2+^. Another report suggested that a membrane potential across the *T. gondii* PV membrane is maintained by proton and potassium P-type ATPases [10]. The limited and, in some cases, apparently discrepant findings for the PV highlight the need to better understand this microenvironment and how it changes during key events in the life cycle.

*T. gondii* persists as a chronic infection in an estimated one third of the global human population, causing opportunistic disease in a subset of those infected. It also produces disease in domestic livestock, wild mammals and birds. In humans, the parasite is especially virulent when acquired congenitally or in reactivated disease, which occurs when the host becomes immune-suppressed. Pathogenesis is driven by iterations of the tachyzoite lytic cycle, which includes host cell invasion, replication within the PV, host cell egress and migration to infect a neighboring cell.

Parasite motility and host cell invasion require the coordinated action of parasite proteins secreted from apical secretory organelles called rhoptries and micronemes. Micronemes are Ca^+2^-regulated secretory organelles that are controlled by phosphorylation-based signaling pathways (reviewed in [11]). Potassium and Ca^+2^ ion fluxes have been shown to influence parasite motility and egress (reviewed in [12]). High K^+^ concentrations, mimicking the intracellular state, inhibit microneme secretion and motility, and a drop in external K^+^ triggers microneme secretion [3]. Although the precise mechanisms of K^+^ sensing by the parasite are still emerging, it is known that intracellular Ca^2+^, phospholipase C and at least two Ca^2+^-dependent protein kinases (CDPKs) are involved [13]. Other studies have shown that Ca^2+^ release from intracellular stores regulates parasite motility by activating the glideosome and apical secretion of transmembrane micronemal adhesins, which engage the motor to transduce power into motion [14]–[16]. Parasite sensing of environmental K^+^ is thought to ensure that the motility system is in neutral during intracellular replication, but is available for engagement to rapidly exit from an infected cell. Malaria sporozoites also respond to K^+^ fluxes [17], implying a conserved mechanism for environmental sensing and regulation of motility.

Earlier investigators of tachyzoite motility additionally found that motility is pH-dependent [18]. Alkaline conditions inhibited motility and acidic buffers induced motility. Here we demonstrate that pH-dependent motility involves the activation of microneme secretion. We also implicate acidification as an enhancer of egress both by promoting microneme secretion and enhancing cytolysis by perforin-like protein 1 (PLP1), a pore forming protein required for efficient egress. Our findings suggest that pH-dependent microneme secretion and activation of PLP1 is another layer of regulating parasite behavior to promote parasite success in changing environments.

## Results

### Effect of pH on microneme secretion and motility

**Figure 1:**
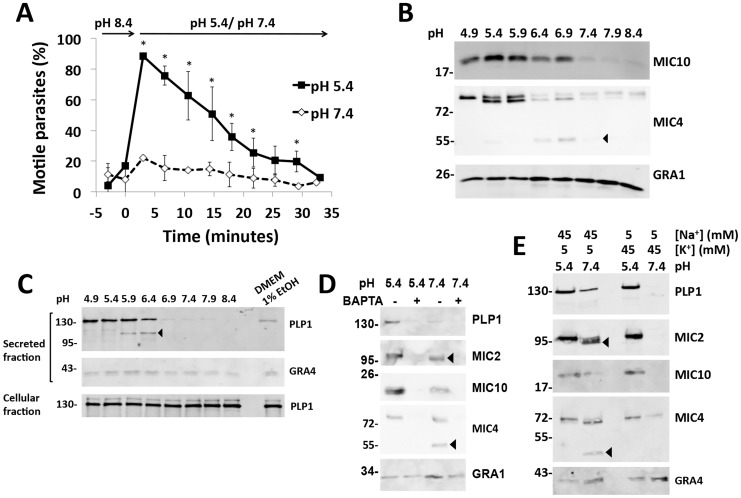
Low pH promotes parasite motility and microneme secretion. (A) Videomicroscopy analysis of pH dependent parasite motility. Parasites were purified in high K^+^ buffer (pH 8.4) and analyzed before and after exchanging to the same buffer at pH 7.4 or 5.4. Graph indicates the average and standard deviation of 3 independent experiments. *p<0.05 vs. pH 7.4 by student's *t*-test. (B) Immunoblot analysis of pH dependent microneme secretion. Parasites were purified in Endo buffer and switched to Endo buffer of the indicated pH, incubated for 2 min at 37°C and placed on ice. Blots of secreted material were probed with antibodies for MIC10, MIC4 or GRA1 as indicated. (C) Low pH induces more microneme secretion than ethanol stimulation and PLP1 is secreted and processed in a pH-dependent manner. Blots of secreted material were probed with antibodies for PLP1 and GRA4 as indicted. Also shown is a blot of PLP1 from cell pellets (cellular fraction) obtained by centrifugation after secretion. (D) Low-pH induced microneme secretion is blocked by the Ca^2+^ chelator, BAPTA-AM, as observed by immunoblot of PLP1, MIC2, MIC10, and MIC4. GRA1 secretion is not affected by BAPTA-AM. (E) Low pH induces microneme secretion in the presence of high K^+^ concentration. Arrowheads for panels B, D and E indicate products of SUB1 proteolysis.

Early studies on tachyzoite motility showed that parasite gliding is inhibited by high concentrations of K^+^ and alkaline pH and promoted by acidic pH [18]. We revisited the effect of pH on gliding by purifying parasites in high K^+^, alkaline buffer (pH 8.4), switching to the same buffer at neutral or low pH and observing motility over time. Low pH stimulated motility in >90% of observed parasites and motility was sustained in a majority of parasites for at least 15 min (Figure 1A), confirming previous findings [18]. Switching from alkaline to neutral pH led to no significant change in motility.

Since parasite motility is linked to microneme secretion, we tested the effect of pH on microneme secretion by incubating parasites in buffer of varying pH and probing the secreted fraction via immunoblots for the microneme adhesive protein MIC2, a galactose-binding protein MIC4 and PLP1. More microneme secretion was detected at low pH compared to neutral and alkaline pH (Figure 1B–C). In contrast, secretion of dense granule proteins GRA1 and GRA4 was largely independent of pH. Low pH induction of microneme secretion was more effective than stimulation with 1% ethanol, which is commonly used to activate microneme discharge (Figure 1C) [19]. Low-pH induced microneme secretion was sensitive to the Ca^2+^ chelator, BAPTA-AM, indicating dependence on intracellular Ca^2+^ (Figure 1D). We also noted pH-dependent differences in the proteolysis of MIC2 and MIC4, which are processed by the micronemal serine protease SUB1. Processing was inhibited by low pH, suggesting the protease SUB1 functions optimally at neutral pH (Figure 1D,E). Acidic pH stimulated microneme secretion despite the presence of high K^+^, whereas minimal secretion occurred at neutral-alkaline pH in a high K^+^ environment (Figure 1E). These findings reveal that low pH can overcome the normally microneme suppressive effects of a high K^+^ environment, which the parasite experiences within an infected cell. The results further suggest that low pH activation of microneme secretion contributes to pH dependent motility.

### Low pH induces parasite egress

**Figure 2:**
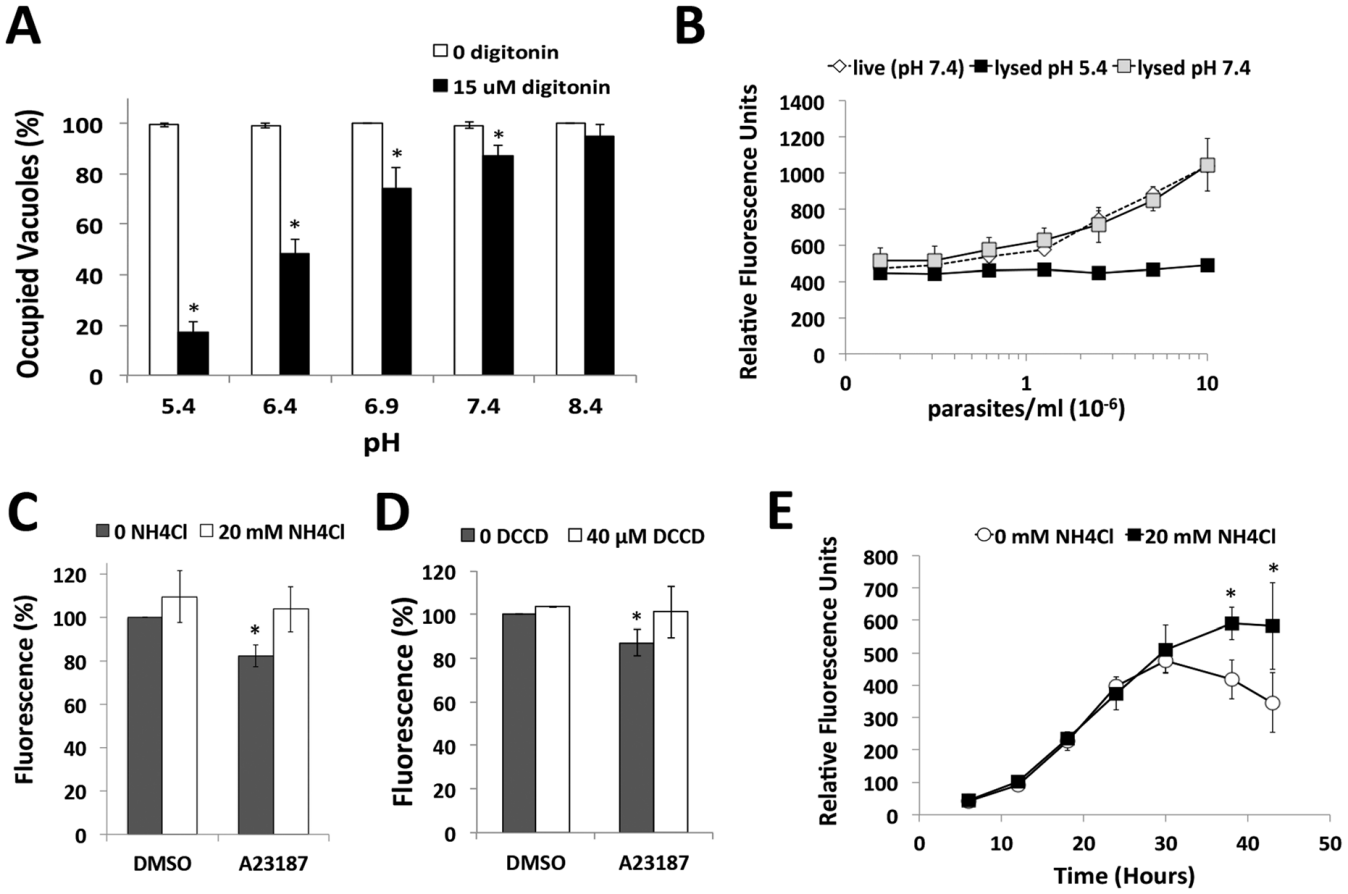
Acidic pH induces parasite egress and pH shifts occur upon egress induction, and late in the replication cycle. (A) Parasite egress quantified by immunofluorescence microscopy. 30 h vacuoles were treated with high K^+^ buffer ±15 µM digitonin at 37°C for 3 min prior to fixation and egress enumeration. *p<0.05 vs. pH 8.4 by student's *t*-test. Graph indicates the average and standard deviation of 3 independent experiments. (B) Superecliptic pHuorin signal is quenched at low pH. HFF cells were inoculated with increasing doses of superecliptic pHluorin expressing *plp1ko* parasites (*plp1ko*sepH) and fluorescence was observed 30 h post-infection in live cells or cells detergent lysed at pH 5.4 or 7.4. (C, D) A moderate decrease in pH occurs during induced egress. HFF inoculated with *plp1ko* parasites expressing superecliptic pHluorin for 30 h before treatment with DMSO or A23187 with or without 20 mM NH_4_Cl (panel C) or 40 µM DCCD (panel D). Results indicate the average and standard deviation of triplicate wells and are representative of 3 independent experiments for NH_4_Cl and 2 independent experiments for DCCD. * *p*<0.05 vs. DMSO by student's *t*-test. (E) pH changes during parasite replication. Fluorescence signal without or with 20 mM NH_4_Cl was followed for *plp1ko* expressing superecliptic pHluorin over the course of intracellular replication. The graph indicates the mean and standard deviation of triplicate wells and is representative of two independent experiments with independent clones. * *p*<0.05 vs. 0 mM NH_4_Cl by student's *t*-test.

Having established that low pH overcomes K^+^ suppression of microneme secretion, we reasoned that exposure of intracellular parasites to low pH should activate microneme secretion and egress despite a high K^+^ environment. We tested this by permeabilizing infected cells with digitonin in high K^+^ buffer with varying pH. Minimal egress was observed under alkaline (pH 8.4) or neutral (pH 7.4) conditions (Figure 2A), consistent with a previous report [3]. On the other hand, acidic pH induced parasite egress in a manner related to the degree of acidification. Together with the above data, these findings suggest that low pH is sufficient to stimulate microneme secretion and initiate motility and parasite egress.

### A shift in pH occurs during induced egress and potentially before or during spontaneous egress

Next we tested the extent to which the pH of the PV changes during induced egress and replication by expressing a pH sensitive GFP variant, superecliptic pHluorin, in the parasitophorous vacuolar space of *plp1ko* parasites (*plp1ko*sepH). *plp1ko* parasites were used instead of WT parasites to avoid losing the probe from the vacuolar space upon secretion of PLP1 during egress. Superecliptic pHluorin is highly fluorescent at neutral pH but is quenched at low pH [20]. We verified the pH-dependence of fluorescence by measuring the signal in superecliptic pHluorin *plp1ko* infected cells lysed at low or neutral pH and in live infected cells. Fluorescence was completely quenched in cells lysed at low pH (Figure 2B). A modest but significant drop in fluorescence was detected upon treating infected cells with Ca^2+^ ionophore (Figure 2C). This decrease in fluorescence was reversed with NH_4_Cl, a weak base, which accumulates in acidic compartments, raising luminal pH. DCCD, a P-type ATPase inhibitor also partially reversed the drop in fluorescence (Figure 2D). These findings suggest a moderate decrease in vacuolar pH occurs upon egress induction. To observe changes in PV pH during parasite replication and spontaneous egress, we measured the fluorescent signal of live superecliptic pHluorin *plp1ko* infected cells over the course of intracellular replication,. If the PV pH is neutral, NH_4_Cl treated cells are expected to have a similar amount of fluorescence as cells in buffer alone. Conversely, if vacuolar pH is acidic, NH_4_Cl treated cells should show a stronger signal than untreated cells due to the pH neutralizing effect of treatment. Super-ecliptic pHluorin signals increased identically during parasite replication until ∼30 h post-inoculation. At this time point, however, the curves began to diverge, with a substantial suppression of fluorescence that was reversed by NH_4_Cl treatment (Figure 2D). Microscopic examination of the infected monolayers indicated that most of the parasites remained intracellular or at least within spherical structures representing failed egress events until after 44 h post-infection. These findings imply a population-scale decrease in vacuolar pH occurs late in the replication cycle, prior to or during spontaneous parasite egress.

### pH-neutralization suppresses parasite egress

**Figure 3:**
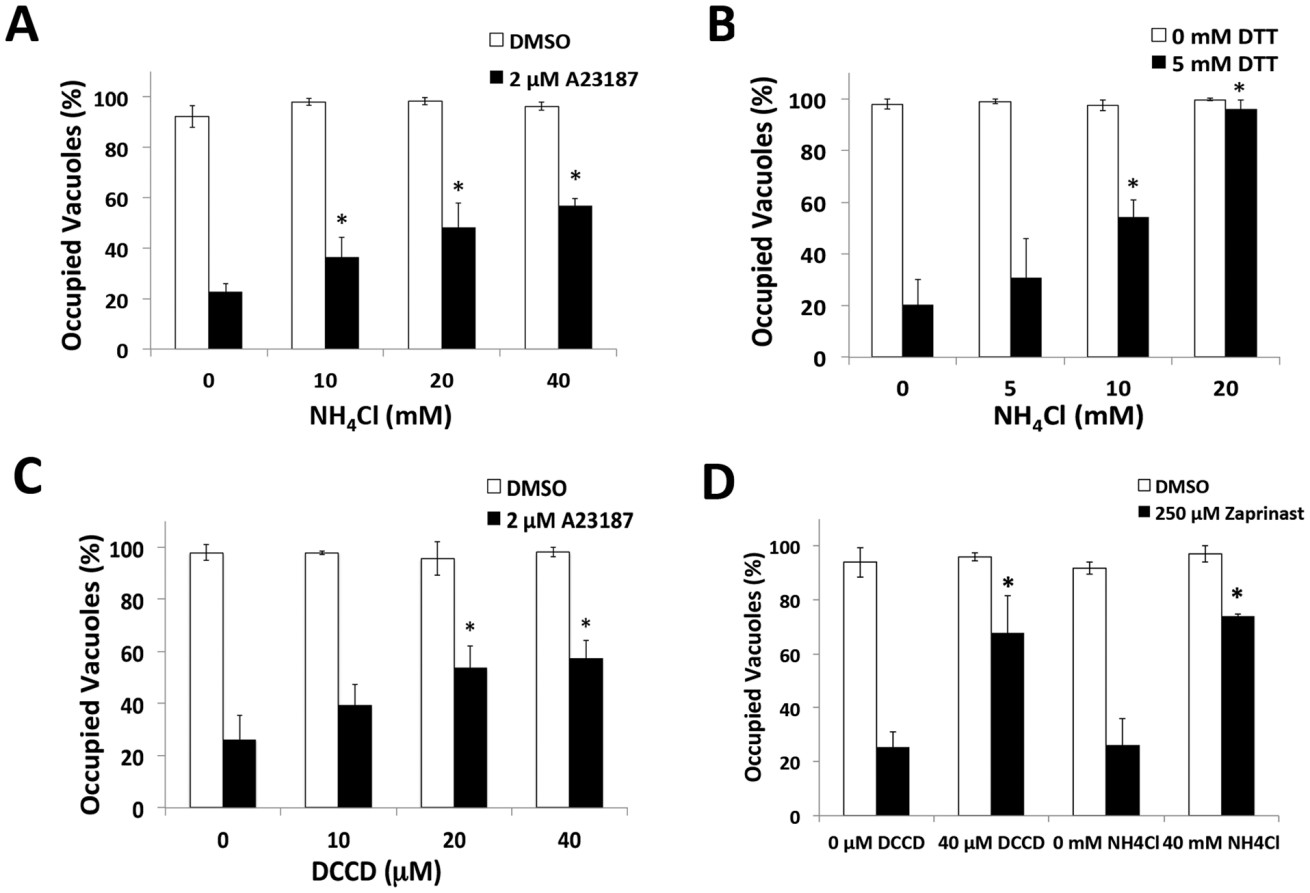
pH neutralization suppresses parasite egress. (A–D) Parasite egress quantified by immunofluorescence microscopy. Wild type (RH) parasites were allowed to replicate for 30 h (A23187, Zaprinast) or 35 h (DTT) prior to pre-treatment with or without inhibitor followed by vehicle (DMSO, buffer) or egress inducer (2 µM A23187, 5 mM DTT, 250 µM Zaprinast) with or without inhibitor for 2 min. Immunofluorescence was performed for parasites (SAG1) and parasitophorous vacuole (GRA7) and occupied vacuoles were quantified. (*p<0.05, student's *t*-test compared to A23187/DTT/Zaprinast alone). Graphs reflect the average and standard deviation of 3 independent experiment; * *p*<0.05 by student's *t*-test vs. A23187/DTT/Zaprinast with no inhibitor.

Next we reasoned if an acidic pH contributes to parasite egress, induced egress should be sensitive to pH neutralization. Consistent with this, we found that parasite egress is suppressed by NH_4_Cl treatment upon stimulation with Ca^2+^ ionophore or dithiothreitol (DTT), an egress inducer that activates a PV nucleotide triphosphatase [5] (Figure 3A–B). PV acidification could occur through passive accumulation of metabolic wastes, or active delivery of protons by a proton-pump. We tested the latter by determining the effect of H^+^-ATPase inhibitors on induced egress. We found that parasite egress was sensitive to the P-type ATPase inhibitor, DCCD (Figure 3C–D), but not the V-type ATPase inhibitor bafilomycin or the H+/K+ exchange inhibitor omeprazole (Figure S1). Egress induced by the phosphodiesterase inhibitor Zaprinast, which triggers microneme secretion and egress via activation of protein kinase G [21], was also sensitive to NH_4_Cl or DCCD treatment (Figure 3D). The above pH-neutralizing agents did not significantly alter motility (Figure S2A, B) or microneme secretion (Figure S2C) induced by the Ca^2+^ ionophore in a neutral buffer, rendering it unlikely that that they affected egress by impairing microneme secretion or the parasite motor system. Together these findings suggest a role for acidic pH and a P-type ATPase in egress.

### pH-neutralization suppresses PLP1 activity during induced egress

**Figure 4:**
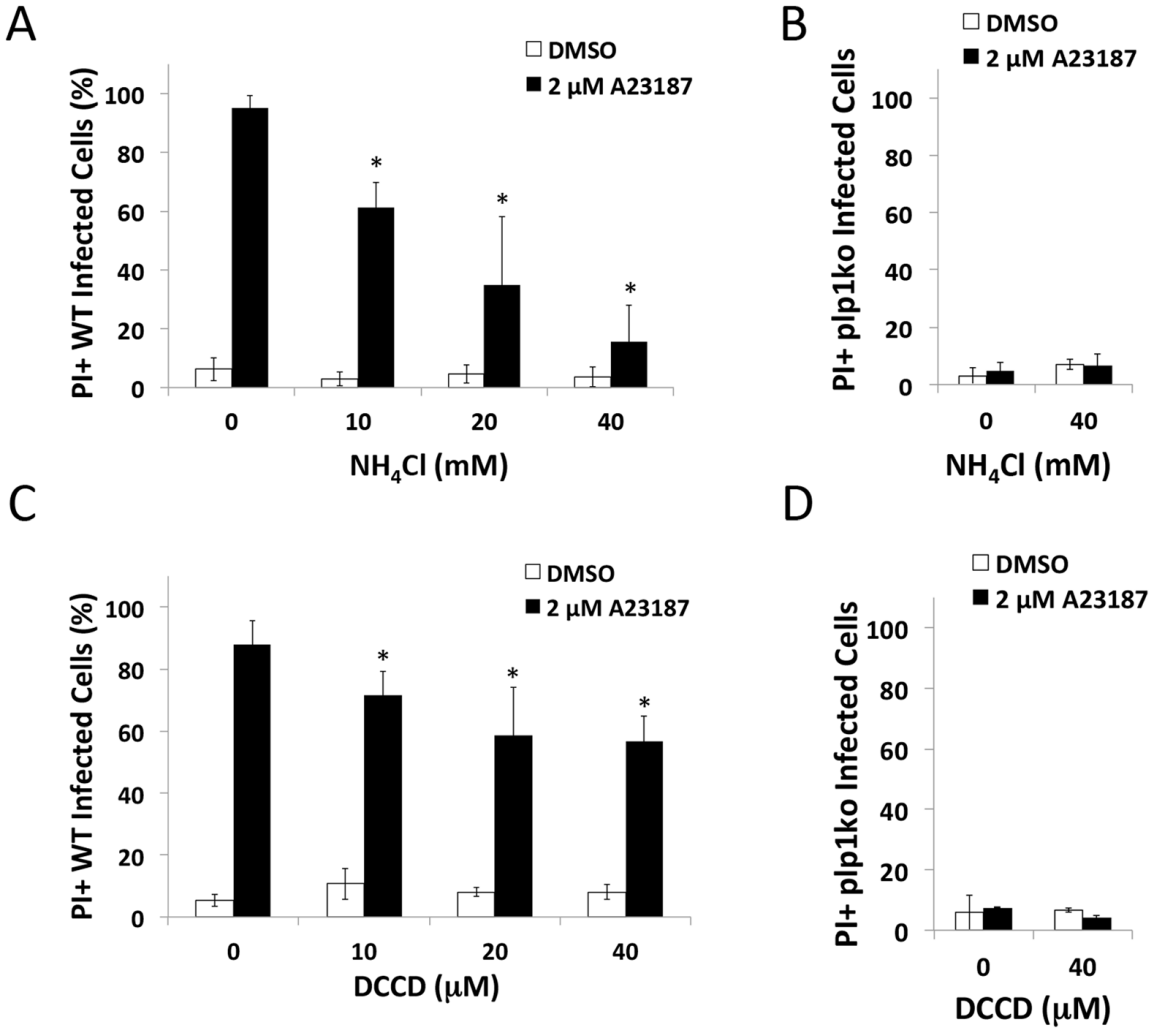
pH-neutralizing agents reduce egress-associated membrane permeabilization. (A–D) RH (WT) or *plp1ko* infected cells were incubated for 30 h prior to treatment with 1 µM CytD with or without inhibitor for 3 min followed by 1 µM CytD with vehicle (DMSO) or ionophore (2 µM A23187) with or without inhibitor plus 12.5 µg/ml propidium iodide (PI) for 3 min. Cells were fixed and stained with DAPI. Infected cells were determined by brightfield and PI positivity by nuclei fluorescent in the red channel. Results indicate average and standard deviation of 3 independent experiments for WT and 2 independent experiments for *plp1ko*. * *p*<0.05 by student's *t*-test.

Next we tested the extent that pH-neutralizing agents affect the activity of important egress effectors during induced egress. Previous work has shown the microneme protein PLP1 to be crucial for rapid parasite egress [22]. To determine whether the reduced parasite egress was due to an inhibition of PLP1 activity occurring with pH-neutralization, we tested the effect of these treatments on egress-associated membrane permeabilization. Since PLP1 is necessary for membrane damage during egress [22], we used the membrane impermeable dye propidium iodide (PI) to assess membrane permeabilization upon treating infected cells with Ca^2+^ ionophore to induce microneme secretion. Parasites were immobilized with the F-actin inhibitor cytochalasin D (CytD) to prevent membrane damage due to actin-myosin dependent parasite motility. Whereas vehicle-treated cells maintained intact membranes, ionophore treatment lead to the permeabilization of the majority of WT-infected cells. This activity was PLP1-dependent since no significant permeabilization was observed in ionophore-treated *plp1ko*-infected cells (Figure 4B, D). Ionophore-induced membrane permeabilization was also sensitive to both NH_4_Cl and DCCD, suggesting that pH-neutralization suppresses PLP1 activity (Figure 4A, C). As indicated above, NH_4_Cl and DCCD did not inhibit parasite gliding motility or microneme secretion, ruling these out processes as possible off-targets of treatment (Figure S2).

### PLP1 cytolytic and membrane binding activity is pH-dependent

**Figure 5:**
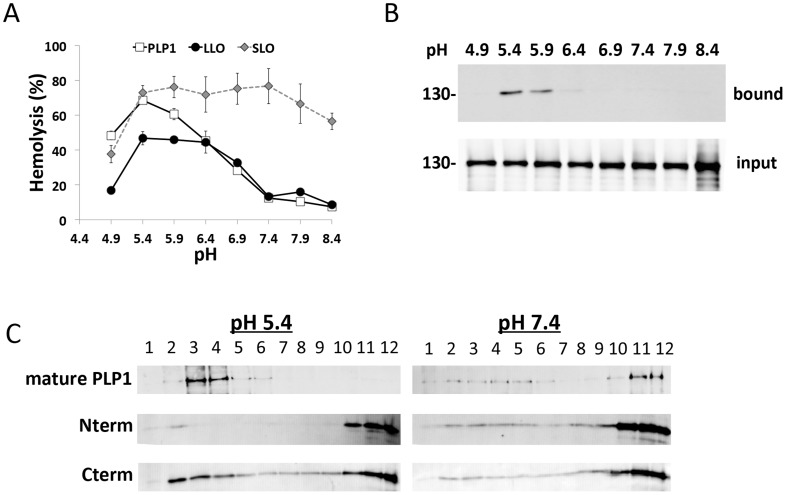
PLP1 displays pH-dependent lytic and membrane binding activity. (A) pH dependent hemolysis. 100 nM recombinant PLP1 or LLO or 2 IU SLO was incubated with sheep erythrocytes before measuring the 450 nm absorbance of the supernatant. Results indicate the average and standard deviation of triplicate wells and the graph is representative of 3 independent experiments. (B) PLP1 binding to membranes is pH dependent. Sheep erythrocyte ghosts were incubated with recombinant PLP1, washed with cold PBS, pelleted, and input and bound fractions were analyzed by immunoblot for PLP1. (C) PLP1 and its Cterm domain display pH dependent membrane binding measured by flotation during sucrose density centrifugation. Recombinant mature, Nterm, and Cterm PLP1 were incubated with sheep erythrocyte ghosts as for B, and the reaction mixture was subjected to sucrose density gradient membrane flotation. Fractions were collected from the top of the sucrose gradient and analyzed by immunoblot. Images are representative of 3 independent experiments.

Since several members of the protein superfamily to which PLP1 belongs are regulated by pH, we used recombinant PLP1 to determine the extent that its activity is pH-dependence. Using hemolysis of erythrocytes as a measure of PLP1 activity, we observed increased PLP1 cytolytic activity beginning at pH 6.4 and peaking at pH 5.4 (Figure 5A). Approximately 7 times more PLP1 activity was seen at pH 5.4 than pH 7.4. The pH dependent profile of PLP1 lytic activity was similar to that of listeriolysin O (LLO), which *Listeria monocytogenes* uses to escape from the acidifying primary vacuole after cell entry [23]. The PLP1 cytolytic profile was distinct from streptolysin O (SLO), which displayed a similar amount of lytic activity across a broad range of pH values. LLO and SLO are cholesterol dependent cytolysins (CDC), which are members of the CDC/membrane attack complex-perforin superfamily that includes PLP1 [24].

We next tested the effect of pH on PLP1 membrane binding using erythrocyte ghosts as a model membrane. We observed that PLP1 membrane binding activity mirrors the lytic activity with more binding at acidic pH than neutral pH (Figure 5B). Previous work demonstrated that PLP1 N- and C-terminal domains both contain membrane-binding activity [25]. Subsequently, we tested for pH-dependent membrane binding of full-length, mature PLP1 and the N-and C-terminal domains by sucrose density gradient membrane flotation. At low pH the majority of mature PLP1 was membrane bound (fractions 1–6), whereas at neutral pH most of the PLP1 remained unbound (fractions 7–12). Membrane binding by the N-terminal domain was not significantly affected by pH, whereas the C-terminal domain showed increased binding at low pH (Figure 5C). These findings are consistent with a previous report showing a dominant role for the C-terminal domain in membrane binding [25]. Together the findings suggest that PLP1 cytolytic activity is pH-dependent at the membrane-binding stage.

### Low pH promotes parasite invasion but also causes PLP1-dependent damage to host cells

**Figure 6:**
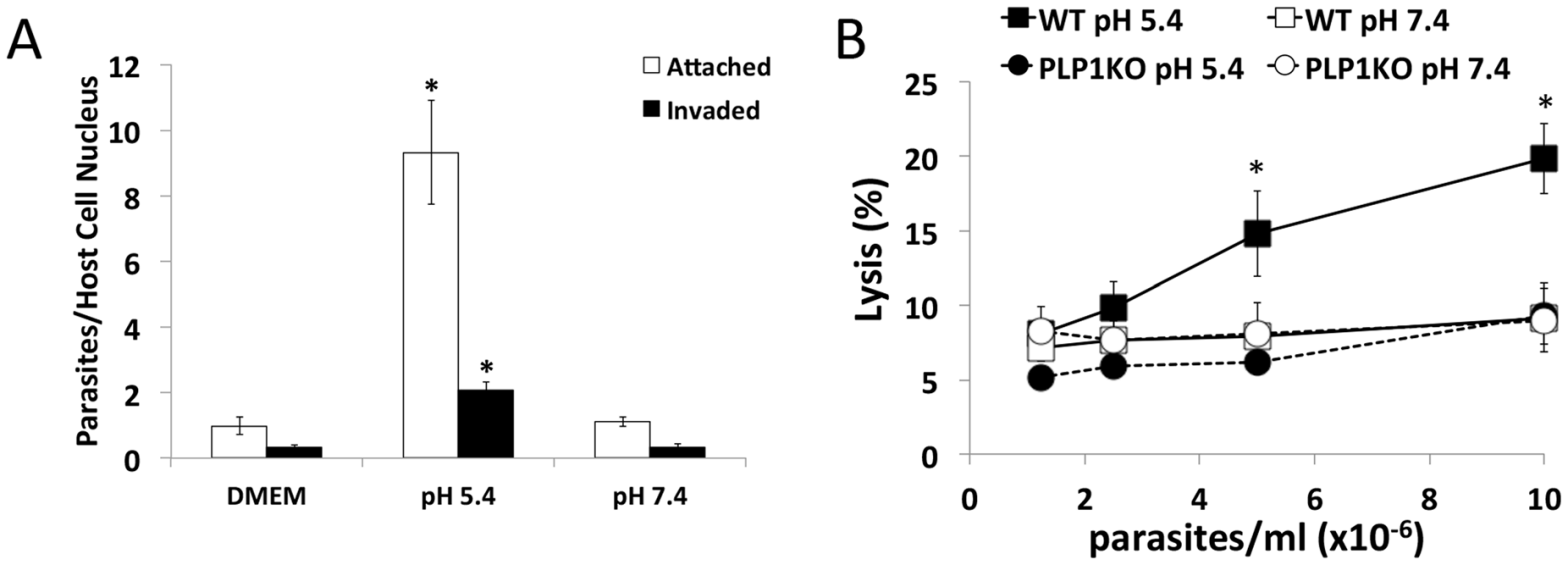
Acidic pH increases invasion but results in host cell damage. (A) Low pH promotes parasite attachment and invasion. HFF cells were pulse invaded for 2 min with parasites in DMEM, or DMEM-like buffer at pH 5.4 or 7.4. *p<0.05 vs. DMEM by student's *t*-test. Graph indicates the average and standard deviation of 3 independent experiments. (B) PLP1- and pH-dependent damage to host cells. HFF cells were preloaded with calcein-AM and WT and *plp1ko* parasites were allowed to settle on host cells in Endo buffer, which was subsequently switched to DMEM-like buffer of the indicated pH and incubated at 37°C for 10 min. Calcein signal in the supernatant was measured by fluorometry. Graph indicates average and standard deviation of triplicate wells and is representative of 3 independent experiments. *p<0.05 vs. WT pH 7.4 by student's *t*-test.

We next investigated the effect of low pH on parasite invasion. Although our above findings predict that the low pH stimulation of microneme secretion should augment parasite invasion, it should also enhance PLP1 activity if invading parasite secretes it. Parasites were purified in high K^+^ buffer and allowed to settle on host cells prior to switching the buffer to either DMEM, or DMEM buffered to pH 5.4 or 7.4 and incubating at 37°C for 2 min, then washed and fixed. We found that low pH substantially increases the amount of attached parasites and invaded parasites (Figure 6A), likely due to increased microneme secretion and motility. Interestingly, acidic conditions appeared to reduce the proportion of invaded parasites relative to attached parasites

To test if PLP1- and pH-dependent membrane damage occurs during invasion we pre-loaded host cells with calcein-AM, and WT and *plp1ko* parasites were allowed to settle on host cells in high K^+^ Endo buffer to block microneme secretion and invasion. Endo buffer was subsequently removed and replaced with DMEM-like buffer at pH 5.4 or 7.4. After 10 minute's incubation, supernatant was collected and calcein release from host cells was quantified by fluorometry. Results were normalized to detergent lysed cells set at 100%. Host cells incubated with WT parasites at neutral pH did not show a density dependent increase in calcein release, consistent with parasite invasion in the absence of overt damage to host cells (Figure 6B). However, host cells incubated with WT parasites at low pH resulted in a significant parasite density-dependent increase in calcein release, a trend that was not seen for *plp1ko* parasites at neutral or acidic pH, indicating a requirement for PLP1 in pH-dependent cell damage. Together these findings suggest that low pH stimulates invasion but also promotes PLP1-dependent wounding of target host cells.

## Discussion

Microneme secretion and motility contribute to host cell invasion and egress, but are quiescent during intracellular replication. Earlier work showed that a high K^+^ environment mimicking the intracellular milieu suppresses parasite motility and egress [3], [18]. A previous report also suggested that *T. gondii* tachyzoite motility is regulated by pH, with moderately acidic conditions strongly enhancing motility [18]. Here we show that an acidic environment promotes microneme secretion, suggesting a mechanism for pH augmentation of motility. We also found that parasite exposure to an acidic environment overcame high K^+^ inhibition of microneme secretion, indicating that acidification of the intracellular environment can trigger microneme secretion and egress even if the high K^+^ intracellular milieu is intact. Consistent with this notion, we found that low pH can drive parasite egress in a high K^+^ environment. Using the pH-sensitive probe superecliptic pHluorin, we observed that egress induction with A23187 leads to a modest decrease in signal, reflecting a shift towards acidic pH. It remains unclear if A23187-induced PV acidification is due to effects of Ca^2+^ flux on proton pumps, due to signaling or secretion events downstream of Ca^2+^ flux, or both. Regardless, using the same probe we also detected a reduction in PV pH late in the replication cycle, possibly near the time of egress. Although these experiments were done with *plp1ko* parasites to minimize loss of the probe from PLP1 membrane damage, experiments showing inhibitory effects of pH neutralization on egress were done with WT parasites, indicating findings are not strain specific.

Earlier investigations determined that upon host cell entry, the PV avoids fusion with endosomes, preventing acidification and degradation of the invaded parasite [26]. Previous efforts to examine vacuolar homeostasis also found a free-flow of small molecules (<1,300 Da) between the PV and host cytosol [7], implying an equivalent neutral pH in both sites. However, these studies were carried out at 24 h or less post-inoculation. Our results concur with the vacuolar pH being neutral at this time point during replication, but suggest that acidification occurs later during the replicative cycle, perhaps immediately prior to egress. Our attempts to directly measure the intravacuolar pH during spontaneous egress were compromised by low expression, poor signal-to-noise and toxicity of ratiometric pHluorin, and difficulties capturing rare egress events with high spatiotemporal resolution. Breaching these obstacles will require substantial improvements in genetically encoded, ratiometric pH biosensors and imaging technologies.

Further support for a role of pH in egress came from showing that treating infected cells with pH-neutralizing agents reduced parasite egress regardless of the egress inducer. Treatment with the P-type ATPase inhibitor, DCCD, impaired induced egress, implying a role for active proton flux during egress. P-type ATPases, also called E1-E2 ATPases, are an evolutionarily conserved group of cation or lipid transporters. The parasite expresses two P-type ATPases (TgPMA1 and TgPMA2) of the Type IIIA subfamily, which includes H^+^-ATPases of plants and fungi [27]. TgPMA1 (TGME49_252640) localizes to the parasite plasma membrane and is substantially upregulated in bradyzoites during the chronic stage of infection [27]. Genetic ablation of *TgPMA1* led to a reduction in tachyzoite to bradyzoite conversion in vitro [27]. TgPMA1 deficient parasites had no reported growth defect as tachyzoites; however, egress was not assessed. TgPMA2 (TGME49_284598) is expressed in tachyzoites and bradyzoites [27], though its function remains to be characterized. The orientation of TgPMA1 and TgPMA2 is such that they are expected to pump protons from the parasite cytoplasm into the PV, consistent with a possible role in acidification of the PV. It is also possible that a DCCD-sensitive host P-type H^+^-ATPase contributes to the putative acidification of the PV. At least one plasma membrane multipass protein is known to occupy the nascent PV membrane after parasite invasion [28], raising the possibility that other such host proteins including ion pumps could reside in the PV membrane. The accumulation of excreted metabolic waste products in the PV or infected cell late during intracellular replication could also contribute to acidification. Also, since it remains unclear how abscisic acid triggers egress, the possibility that it contributes to PV acidification warrants consideration.

Other pore forming proteins including LLO are regulated by pH. LLO pH-sensitivity is mediated by three acidic amino acids in the transmembrane helices, which are part of domain 3 [29], [30]. These residues are thought to repel one another at neutral pH leading to protein denaturation and loss of activity. Our findings suggest that PLP1 uses a distinct mechanism, however, since low pH augments PLP1 membrane binding via the C-terminal domain, which is positionally and functionally equivalent to domain 4 of LLO. Although the N-terminal domain also has membrane-binding activity, its membrane binding was not significantly affected by pH. Our findings also indicate that PLP1 membrane binding is pH-dependent, but it remains possible that subsequent steps such as oligomerization and membrane insertion are also pH regulated. The pore forming activity of human perforin is pH-dependent at a step following membrane binding [31]. Thus, pore-formation may be regulated by different environmental conditions at multiple steps of pore-formation. Future structural comparisons of PLP1 with LLO and perforin might illuminate divergent features that modulate the activity of these proteins in the varied environments in which they function.

The increased microneme secretion at low pH also leads to dramatically higher parasite attachment and invasion. However, this augmentation of parasite association with the host cell comes at the expense of increased cell wounding. It is difficult to distinguish the extent to which increased microneme secretion versus increased PLP1 activity contributes to the membrane damage observed. The effect is probably due to a combination of the two factors. Regardless, membrane permeabilization was entirely PLP1-dependent since it was not detected in the PLP1-deficient strain at either pH.

Collectively, our findings suggest a working model in which acidification of the PV during late stage parasite replication or immediately preceding egress augments both microneme secretion and PLP1 activity. That pH 5.9–6.4 is sufficient to promote microneme secretion and PLP1 membrane binding implies that moderate acidification is adequate to enhance microneme- and PLP1-dependent egress. PV acidification is capable of overcoming K^+^-dependent suppression of microneme secretion and motility, thus it can act as a primary trigger in the high K^+^ intracellular environment of a viable cell. Initial pH dependent release of PLP1 is expected to cause membrane damage resulting in a loss of K^+^ from the infected cell, thus further promoting Ca^2+^signaling, motor activation and microneme secretion to accelerate membrane damage, motility and egress. It should be noted, however, that while motility independent disruption of the PVM during egress is strictly PLP1-dependent [22], the contribution of PLP1 to disruption of the host plasma membrane remains unknown. The model further predicts that the low K^+^, neutral pH environment experienced by extracellular parasites after egress augments microneme secretion but suppresses PLP1 activity. Thus the extracellular environment is conducive to microneme-based motility, parasite attachment and invasion, but it suppresses PLP1-dependent damage to the membrane of the target cell, thereby permitting correct formation of the PV during invasion. This model does not exclude other levels of regulation e.g., differential accessibility of PLP1 receptors or functionally distinct subpopulations of micronemes [32], which could occur in parallel to ensure maximal PLP1 activity during egress while minimizing membrane damage during invasion. It is also expected that the proposed pH regulatory mechanism functions in parallel with other sensory and signaling pathways to coordinate egress under different circumstances.

## Materials and Methods

### Statistics and experimental replicates

Student's t-tests were used to assess differences in quantitative experiments, which were performed at least three times, with technical replicates within each experiment in some cases. Qualitative experiments were performed at least twice and often three to four times.

### Parasite propagation

*T. gondii* tachyzoites were maintained in human foreskin fibroblasts (HFF) as previously described [33].

### Gliding assays

Gliding experiments were conducted using a Zeiss temperature/CO_2_/humidity modulation system on a Zeiss Axio inverted microscope equipped with an Axiocam MRM CCD camera. For pH-dependent gliding, parasites were filter purified in high K^+^ buffer (145 mM KCl, 5 mM NaCl, 1 mM MgCl_2_, 15 mM MES, 15 mM HEPES, pH 8.4) and allowed to settle in a glass-bottom petri dish. After parasites had settled, images were collected every 100 ms for 6 min. Then buffer was exchanged for the same buffer adjusted to pH 7.4 or 5.4 and images were collected every 100 ms for 30 min. For inhibitor gliding experiments, parasites were filter-purified in PBS, resuspended and allowed to settle in HBSSC (Hanks buffered salt solution, 10 mM HEPES, 1 mM CaCl_2_, 1 mM MgCl_2_). After initial images were collected, buffer was exchanged for 40 mM NH_4_Cl or 40 µM DCCD in HBSSC and parasite motility was observed as above. Maximum projection images and videos were examined for motile parasites and the percent of motile parasites graphed over time. For inhibitor treatment assays, values were normalized to the percent motile parasites at time zero and the fold change in motility over time was graphed.

### Microneme secretion assays

Microneme secretion induced with A23187 or ethanol as described previously [14] in the presence of vehicle, 10, 40 mM NH_4_Cl, or 10, 40 µM DCCD [10]. Low-pH induced secretion was tested by purifying parasites in Endo buffer (44.7 mM K_2_SO_4_, 106 mM sucrose, 10 mM MgSO_4_, 20 mM Tris-H_2_SO_4_ (pH 8.2), 5 mM glucose, 3.5 mg/ml BSA) [18], and re-suspending in invasion buffer (110 mM NaCl, 0.9 mM NaH_2_PO_4_, 44 mM NaHCO_3_, 5.4 mM KCl, 0.8 mM MgSO_4_, 1.8 mM CaCl_2_) or Endo buffer of the indicated pH at 37°C for 2 min. Calcium-dependence of secretion was tested by pre-incubation with BAPTA-AM as described previously with the following modifications: freshly egressed RH parasites were purified in Endo buffer and pre-treated with BAPTA-AM in Endo buffer at 37°C, pelleted, and then resuspended in 37°C Endo buffer of pH 5.4 or 7.4 with or without BAPTA-AM, incubated for 2 min at 37°C and then placed on ice [14]. To test the effect of potassium inhibition of microneme secretion, freshly egressed RH parasites were purified in Endo buffer at room temperature, pelleted, and resuspended in 37°C Endo buffer with either 45 mM KCl/5 mM NaCl or 5 mM KCl/45 mM NaCl, incubated at 37°C for 2 min, and then placed on ice. For all secretion assays, after incubating on ice, parasites were pelleted at 4°C, the secreted fraction (supernatant) was removed and spun again and the parasite pellet was washed in cold PBS prior to resuspending in boiling SDS-PAGE loading buffer. Secreted fractions and parasite pellets were examined by immunoblotting with the indicated antibodies.

### Digitonin permeabilization assay

Low-pH induced egress was tested by inoculating HFF in an 8-well chamber slide with 3 µl of freshly egressed RH parasites/well and incubating for 30 h. The slide was then washed twice with warm high K^+^ buffer (pH 8.4) and the buffer was replaced with buffer of the same composition and varying pH with and without 15 µM digitonin. The slide was incubated at 37°C for 3 min, fixed with 8% formaldehyde and occupied vacuoles were enumerated as previously described [25].

### pHluorin assays

Superecliptic and ratiometric pHluorin vectors were kindly provided by Dr. Gero Miesenbock by material transfer agreement (University of Michigan, SSP no. 13477; Memorial Sloan-Kettering Institute, SK# 19367) [20]. The genes were subcloned into the DsRed vacuolar expression vector [22]. Parasites were transfected with plasmid, transformed parasites were selected for with chloramphenicol and cloned by limiting dilution. Superecliptic pHluorin was highly expressed by parasites in the PV.

pH-sensitivity was tested by inoculating HFF with varying concentrations of super-ecliptic pHluorin expressing *plp1ko* parasites (*plp1ko*sepH) in a 96-well plate, and incubating for 30 h at 37°C. *plp1ko* parasites were used to retain the fluorescent signal in the parasitophorous vacuole upon egress induction. Wells were washed twice with warm PBS and PBS without Triton-X-100 was used to measure fluorescence in live, infected cells. PBS (pH 5.4 or 7.4) with 0.1% Triton-X-100 was used to measure fluorescence in lysed cells.

A23187-induced changes in superecliptic pHluorin signal in live, infected cells were tested by inoculating HFF (grown in phenol red-free DMEM) in a 96-well plate with *plp1ko*sepH parasites (2.5×10^6^ parasites/ml, 100 µl/well), incubating for 30 h, washing the wells twice with warm PBS, and adding HBSSC with 2 µM A23187 or DMSO, without or with 20 mM NH_4_Cl or 40 µM DCCD. The time between the addition of the compounds and fluorescence measurement was approximately 5 min. The majority of *plp1ko* parasites are unable to egress in this time period.

Fluorescence over time in live, infected cells was observed by inoculating HFF with *plp1ko*sepH (1×10^5^ parasites/ml, 100 µl/well) in a 96-well plate. At the indicated time points, 2 sets of triplicate wells were washed twice with warm PBS. One hundred µl warm HBSSC with or without 20 mM NH_4_Cl was added and fluorescence was read in a pre-warmed plate reader. Following the fluorescence reading, the plates were reincubated and a new set of wells was used for each time point. At each time point, wells were briefly examined microscopically prior to the fluorescent reading to check if the majority of parasites were intracellular. The experiment was terminated beyond 44 h due to the progression of endogenous egress.

Fluorescence was measured at excitation 485/20 nm and emission 530/25 nm in a BioTek Synergy HT microplate reader at 37°C. Background from uninfected cells was subtracted from the total fluorescence for each condition. Data points represent the average and standard deviation of 2 or 3 independent experiments consisting of triplicate wells in each experiment. All assays were conducted in clear plastic 96-well plates since optimization experiments indicted they performed equally well as black-sided well plates.

We also attempted to measure the pH of the PV using ratiometric pHluorin [20]. Transfection of the original ratiometric pHluorin sequence in the DsRed vacuolar expression vector [22] into *T. gondii* tachyzoites followed by drug selection and isolation of clones revealed that ratiometric pHluorin was transcribed but not translated as demonstrated by production of mRNA by RT-PCR and lack of detection by fluorescence microscopy, immunoblot or pulse chase ^35^S-methionine/cysteine metabolic labeling and immunoprecipitation. Ratiometric pHluorin was subsequently codon-optimized, chemically synthesized (GenScript Inc) and subcloned into the DsRed vacuolar expression vector as above. Codon-optimized ratiometric pHluorin was expressed by the parasites, and fluorescent parasites recovered upon drug selection and cloning by limited dilution. Imaging was performed with Zeiss filter sets 21 HE (excitation 340/30 nm +387/15 nm, emission 510/90 nm) and 38 HE (excitation 470/40 nm, emission 525/50 nm). Fluorescent parasites were lost upon prolonged passage despite continuous drug selection, thus limiting the experiments to transiently transfected parasites. However, due to low expression and fluorescence, the exposure times required for signal detection were longer than those needed to measure rapid changes in pH during induced egress. Additionally, PVs of mock transfected parasites were noted to have varying degrees of autofluorescence in the 390 nm channel, which is the pH-sensitive wavelength, giving low confidence in the ability of ratiometric pHluorin to accurately reflect changes in PV pH especially as the total amount of signal was low late in the endogenous replication cycle.

### Egress assays and egress-associated permeabilization of the parasitophorous vacuole membrane

Egress assays with A23187 were conducted as previously described [33] with the following modifications: HFF in an 8-well chamber slide were inoculated with 3 µl of freshly egressed wild type (RH) parasites and incubated for 30 h at 37°C. Wells were washed twice with warm PBS and a 2 minute pretreatment at 37°C of the inhibitor (NH_4_Cl, bafilomycin, dicyclohexylcarbodiimide (DCCD), omeprazole) in HBSSC was applied prior to addition of 4 µM A23187 (2 µM final concentration) or DMSO with or without the indicated treatment in HBSSC for 2 minutes and fixation in 8% formaldehyde. Inhibitors were purchased from Sigma and tested at the indicated concentrations. Zaprinast-induced egress was tested at 250 µM (final concentration) in the same manner as A23187-induced egress. Immunofluorescence was performed and enumerated for SAG1 and GRA7 and occupied/unoccupied vacuoles as previously described [33].

Egress-associated membrane permeabilization was tested by inoculating HFF cells in an 8-well chamber slide with 2 µl of freshly egressed RH or *plp1ko* parasites and incubating for 30 h at 37°C. Following 2 washes with warm PBS, wells were treated with 100 µl HBSSC+1 µM CytD with or without NH_4_Cl or DCCD for 3 min at 37°C. Then 100 µl/well was added of 4 µM A23187/DMSO, 1 µM CytD, 12.5 µg/ml propidium iodide (PI) with the indicated final concentrations of NH_4_Cl and DCCD and incubated for 3 min. Following the incubation, cells were washed twice with warm PBS, fixed with 4% formaldehyde and stained with DAPI. Membrane permeabilization was quantified by the number of infected cells with PI-positive nuclei.

### Recombinant protein expression and assays

Recombinant PLP1 and LLO were generated as previously described [33]. Streptolysin O (SLO) was handled according to the manufacturer's instructions (Murex Diagnostics). pH-dependent hemolysis was assessed by washing erythrocytes in PBS (pH 7.4), pelleting the RBC, and re-suspending in PBS of indicated pH (prepared by mixing sodium mono- and diphosphate in different amounts and adjusting pH with HCl or NaOH) with 100 nM recombinant protein. RBC and recombinant protein were incubated at 37°C for 1 h, pelleted, and hemolysis was measured by absorbance at 540 nm of the supernatant.

pH-dependent binding was tested by incubating erythrocyte ghosts, prepared as previously reported, with recombinant protein in PBS of the indicated pH [33]. RBC ghosts and recombinant protein was incubated at 37°C for 30 min; cells were pelleted and washed three times with cold PBS at neutral pH and bound samples were analyzed by SDS-PAGE and immunoblot.

### Invasion assays

Parasite invasion was tested as previously described [34] with the following modifications: parasites were purified in Endo buffer and allowed to settle on HFF in an 8-well chamber slide at room temperature for 20 min. Then Endo buffer was replaced with either DMEM or DMEM-like buffer (invasion buffer) (110 mM NaCl, 0.9 mM NaH_2_PO_4_, 44 mM NaHCO_3_, 5.4 mM KCl, 0.8 mM MgSO_4_, 1.8 mM CaCl_2_) at pH 5.4 or 7.4 and the slide was incubated at 37°C for 2 min. The buffer or media was removed, wells were washed twice with room temperature PBS and the slide was fixed with 0.4% formaldehyde in PBS. Immunofluorescence staining and parasite quantification was conducted as previously described for attached and invaded parasites.

### Cell-wounding assays

Cell wounding was tested by pre-loading host cells with 1 µM calcein-AM in phenol-red free DMEM and incubating for 1 h at 37°C, followed by two washes with warm PBS. Parasites were filter-purified in Endo buffer and applied to host cells in a 96-well plate by centrifuging at 500 g for 5 min. Supernatant was removed and replaced with 100 µl/well DMEM-like buffer, pH 7.4 or 5.4. Plates were incubated for 10 min at 37°C and centrifuged as above. Fifty µl of supernatant was transferred to another plate and calcein fluorescence was read in a 96-well plate reader.

## Supporting Information

Figure S1 **Bafilomycin does not inhibit rapid parasite egress.** Parasites egress was quantified by immunofluorescence microscopy. Parasites were allowed to replicate for 30 h prior to treatment with vehicle (DMSO, buffer) or egress inducer (2 µM A23187) for 2 min in the presence of the indicated concentration of bafilomycin. Immunofluorescence was performed for parasites (SAG1) and parasitophorous vacuole (GRA7) and occupied vacuoles were quantified. Results are the average of 3 independent experiments.
(TIF)

Figure S2 **pH-neutralizing treatments do not affect parasite motility or microneme secretion.** (A,B) Parasite motility was observed by light videomicroscopy in vehicle and with the indicated treatments. Values are normalized to the percent motile parasites at time zero. The graph indicates the average and standard deviation over three independent experiments. (C) Microneme secretion of extracellular parasites was induced with A23187 with vehicle or one of the indicated compounds at a low or high concentration (10, 40 mM NH_4_Cl; 10, 40 µM DCCD) for 2 min and the secreted fraction was examined by immunoblot. (D) Overnight replicated parasites were immobilized with 1 uM cytD for 10 min prior to treatment with vehicle (DMSO) or stimulation with A23187 alone or with 40 mM NH4Cl or 40 µM DCCD for 2 min. Samples were fixed with paraformaldehyde, semipermeablized with saponin and immunofluorescence stained for SUB1 (arrow) on the surface of parasites. Scale bar, 5 µm.
(TIF)
