# Supplementary figures and images for: Acidification Activates Toxoplasma gondii Motility and Egress by Enhancing Protein Secretion and Cytolytic Activity
Source: PLoS Pathog. 2014 Nov 6;10(11):e1004488. doi: 10.1371/journal.ppat.1004488 (PMC4223073; doi:10.1371/journal.ppat.1004488)

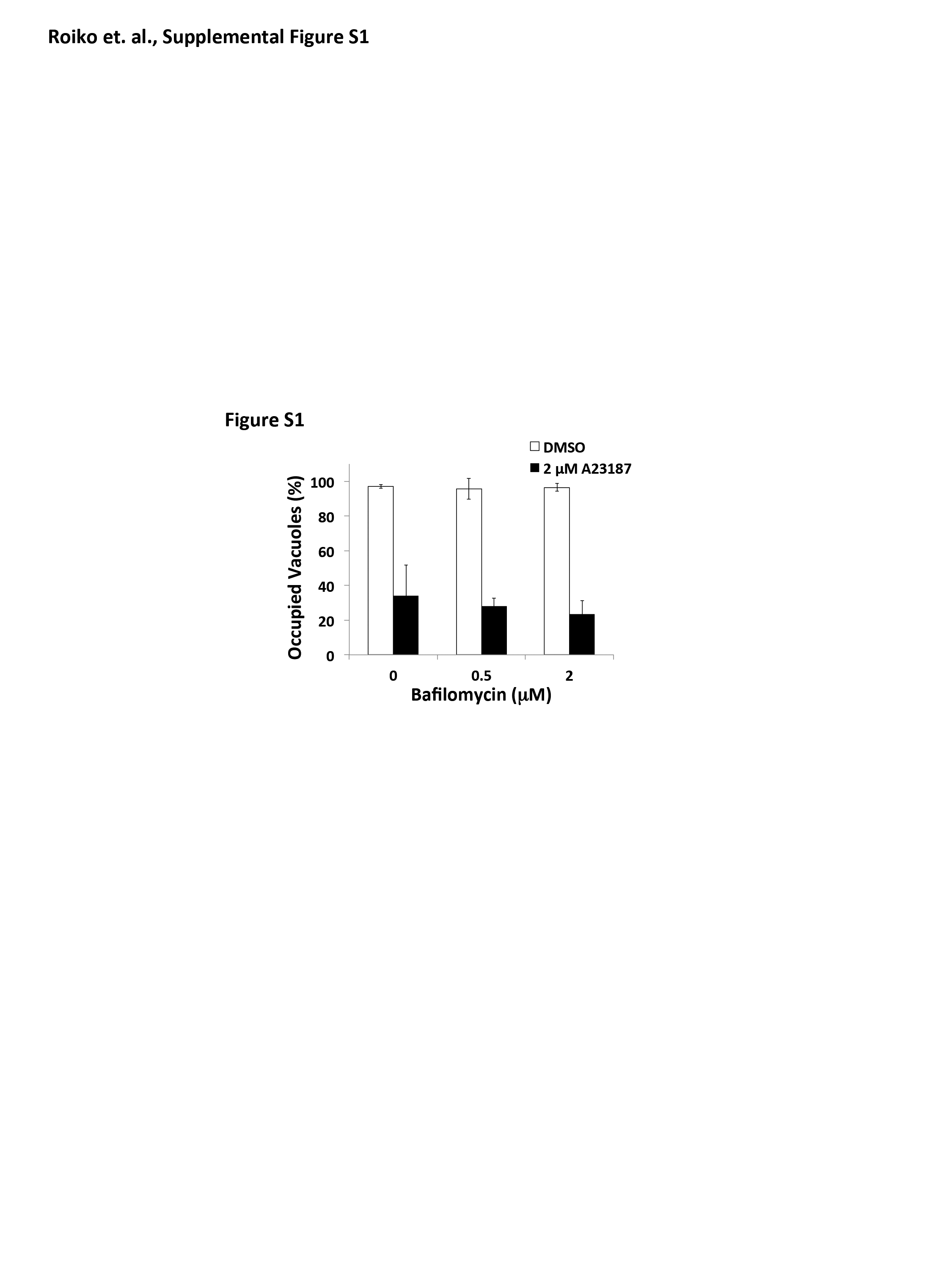

Supplement: Figure S1 — Bafilomycin does not inhibit rapid parasite egress. Parasites egress was quantified by immunofluorescence microscopy. Parasites were allowed to replicate for 30 h prior to treatment with vehicle (DMSO, buffer) or egress inducer (2 µM A23187) for 2 min in the presence of the indicated concentration of bafilomycin. Immunofluorescence was performed for parasites (SAG1) and parasitophorous vacuole (GRA7) and occupied vacuoles were quantified. Results are the average of 3 independent experiments. (TIF) [file ppat.1004488.s001.tif]

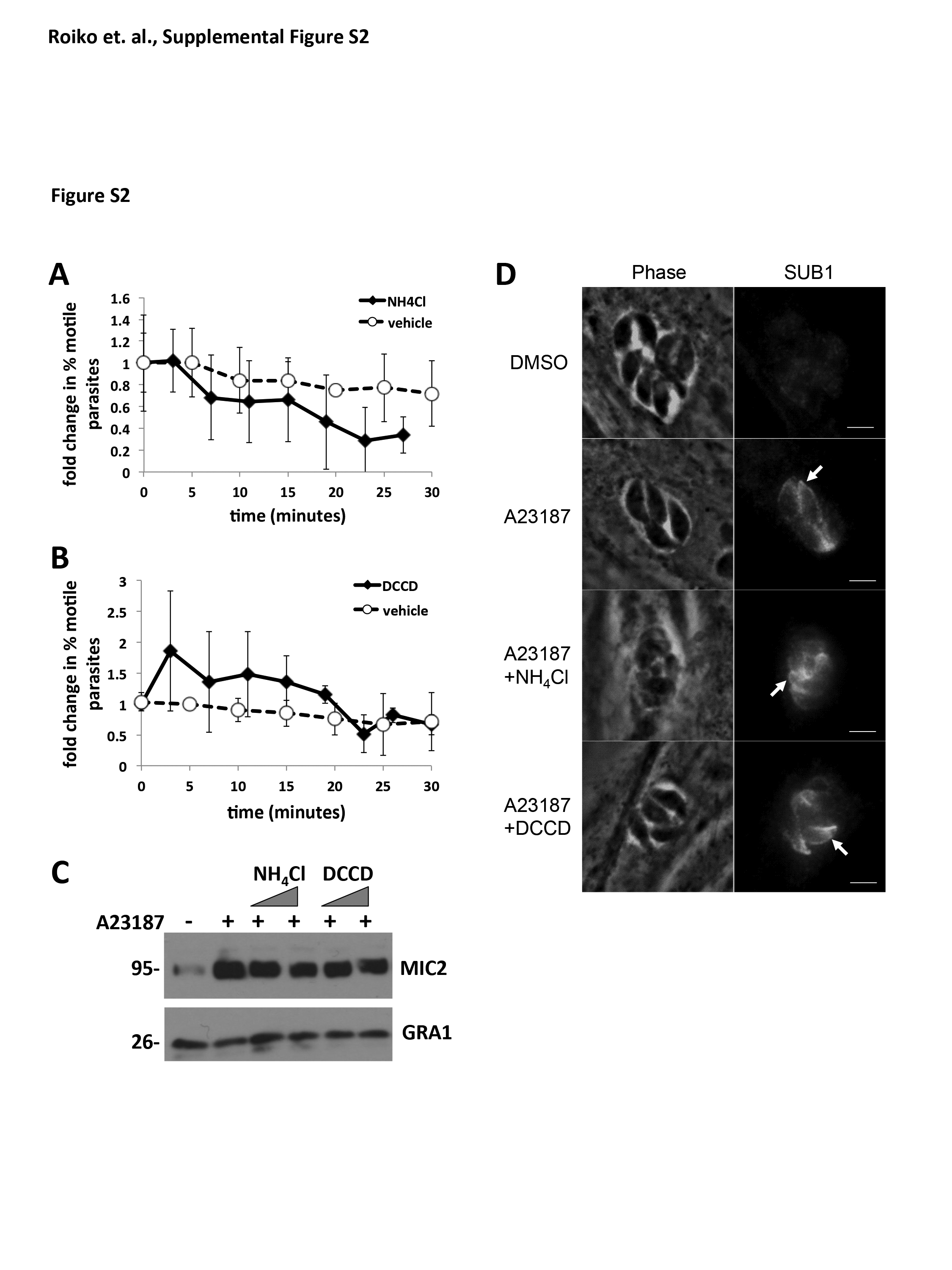

Supplement: Figure S2 — pH-neutralizing treatments do not affect parasite motility or microneme secretion. (A,B) Parasite motility was observed by light videomicroscopy in vehicle and with the indicated treatments. Values are normalized to the percent motile parasites at time zero. The graph indicates the average and standard deviation over three independent experiments. (C) Microneme secretion of extracellular parasites was induced with A23187 with vehicle or one of the indicated compounds at a low or high concentration (10, 40 mM NH4Cl; 10, 40 µM DCCD) for 2 min and the secreted fraction was examined by immunoblot. (D) Overnight replicated parasites were immobilized with 1 uM cytD for 10 min prior to treatment with vehicle (DMSO) or stimulation with A23187 alone or with 40 mM NH4Cl or 40 µM DCCD for 2 min. Samples were fixed with paraformaldehyde, semipermeablized with saponin and immunofluorescence stained for SUB1 (arrow) on the surface of parasites. Scale bar, 5 µm. (TIF) [file ppat.1004488.s002.tif]
